# Gigahertz thermoelastic acousto-optic modulation in lithium niobate integrated photonic device

**DOI:** 10.1515/nanoph-2025-0252

**Published:** 2025-09-30

**Authors:** Zheng Zheng, Hanke Feng, Ahmet Tarık Işık, Peter J.M. van der Slot, Cheng Wang, David Marpaung

**Affiliations:** Nonlinear Nanophotonics Group, MESA+ Institute for Nanotechnology, University of Twente, Enschede, The Netherlands; City University of Hong Kong, Hong Kong, China

**Keywords:** acousto-optic modulation, surface acoustic waves, thin-film lithium niobate, optomechanics

## Abstract

Tailoring the interaction between acoustic waves and optical waves in integrated photonic circuits has emerged as a promising avenue for quantum optics and high-speed information processing. Among various approaches, on-chip acousto-optic modulation (AOM) has been extensively explored in the past decade and successfully demonstrated across many integrated photonics platforms. In this paper, we design and fabricate metallic gratings with periods varying from 0.4 μm to 3 μm deposited on a half-etched thin film lithium niobate (TFLN) device cladded by 1-μm thick silica. By illuminating the metallic grating with an intensity-modulated pump beam, we can generate thermoelastically driven SAWs with frequencies up to 6.58 GHz, and a linewidth of 1.8 MHz at a center frequency of 1.41 GHz. This thermoelastic approach eliminates reliance on a piezoelectric material response, offering broader compatibility for integrated photonics applications.

## 1 Introduction

Efficient and flexible acousto-optic modulation (AOM) in chip-scale integrated photonics can provide substantial benefits for signal processing, optical communications [1], [2], [3], [4], [5], [6], [7], [8], [9], and potentially quantum photonics [10], [11]. One of the key requirements for an efficient on-chip AOM is a large acousto-optic spatial overlap. However, coherent spatial control of acoustic waves and optical waves in on-chip AOM is not straightforward, especially in waveguide layer stack structures. The acoustic velocities in most core materials for optical waveguides are higher than that in commonly used silica cladding, leading to leaky acoustic waves and, therefore, limiting the on-chip AOM efficiency. One solution, a suspended waveguide in air [6], [7], [10], [11] has been demonstrated but makes it more difficult to support large-scale integration. Another solution is to generate surface acoustic waves (SAWs) and locate the optical waveguide near [1], [12], [13] or on the surface [2], [3], [4], [5], [6], [14], increasing the interaction area between acoustic waves and optical waves to achieve efficient on-chip AOM.

SAW-based AOM has been successfully demonstrated in many integrated photonic platforms, mainly in piezo-electric materials, such as lithium niobate (LN) [2], [3], [4], [5], [14], aluminum nitride (AlN) [10], and gallium arsenide (GaAs) [7]. These platforms have a wide transparency range and also support electrical–mechanical energy conversion via applying oscillating voltages on the electrodes of a so-called interdigital transducer (IDT). By engineering the optical waveguide structure and the IDT design, on-chip AOM has been achieved up to several gigahertz, which is interesting for integrated microwave photonics [15], [16], [17], [18].

In other low-loss platforms without piezoelectricity, which include silicon (Si) and silicon nitride (Si_3_N_4_), AOM cannot be achieved directly for now. Hence, on-chip AOM in these platforms are achieved by cointegrating a piezo-electric layer on top of them, either through wafer bonding [19] or through deposition [1], [6], [12], [13], [20], [21]. Therefore, the excited SAW in the piezo-layer penetrates through the top cladding and interacts with the optical waves propagating in an optical waveguide nearby to the surface, but increase the optical propagation loss by a factor of 4 [19]. In both cases, IDT impedance matching is inevitable, and the current leads between IDT and contact pads make the IDT more susceptible to electromagnetic interference, i.e., picking up undesired electromagnetic signals.

Instead of electrically driven SAW, we consider thermally driven SAW [22], [23], [24] to realize acousto-optic interactions compatible with various low-loss photonic platforms. Such an alternative technique may also be beneficial for piezo-active photonic materials, e.g., AlN and LN, because the optical driving signal can be routed on the same photonic chip, thereby freeing up the area for routing electric signals. Meanwhile, this technique is not sensitive to or producing electromagnetic interference. So far, this technique has been demonstrated on silicon on insulator (SOI) platform [22], [23], and, for the first time, we optimize this technique for the silica-cladded thin film lithium niobate (TFLN) platform.

In this paper, we demonstrate thermoelastic-generated SAW with frequencies up to 6.58 GHz using a 50-nm thick gold metal grating located on a 1-μm thick silica top cladding. To measure this SAW, we demonstrate optical phase modulation in a half-etched TFLN optical waveguide, employing the SAW emitters on the side. The linewidth of the generated SAW is 1.8 MHz for an acoustic center frequency of 1.41 GHz, corresponding to a 2-μm period of the grating. We quantitatively discuss the thermoelastic process and propose some strategies to improve the AOM efficiency in the future.

## 2 Design and fabrication

**Figure 1:**
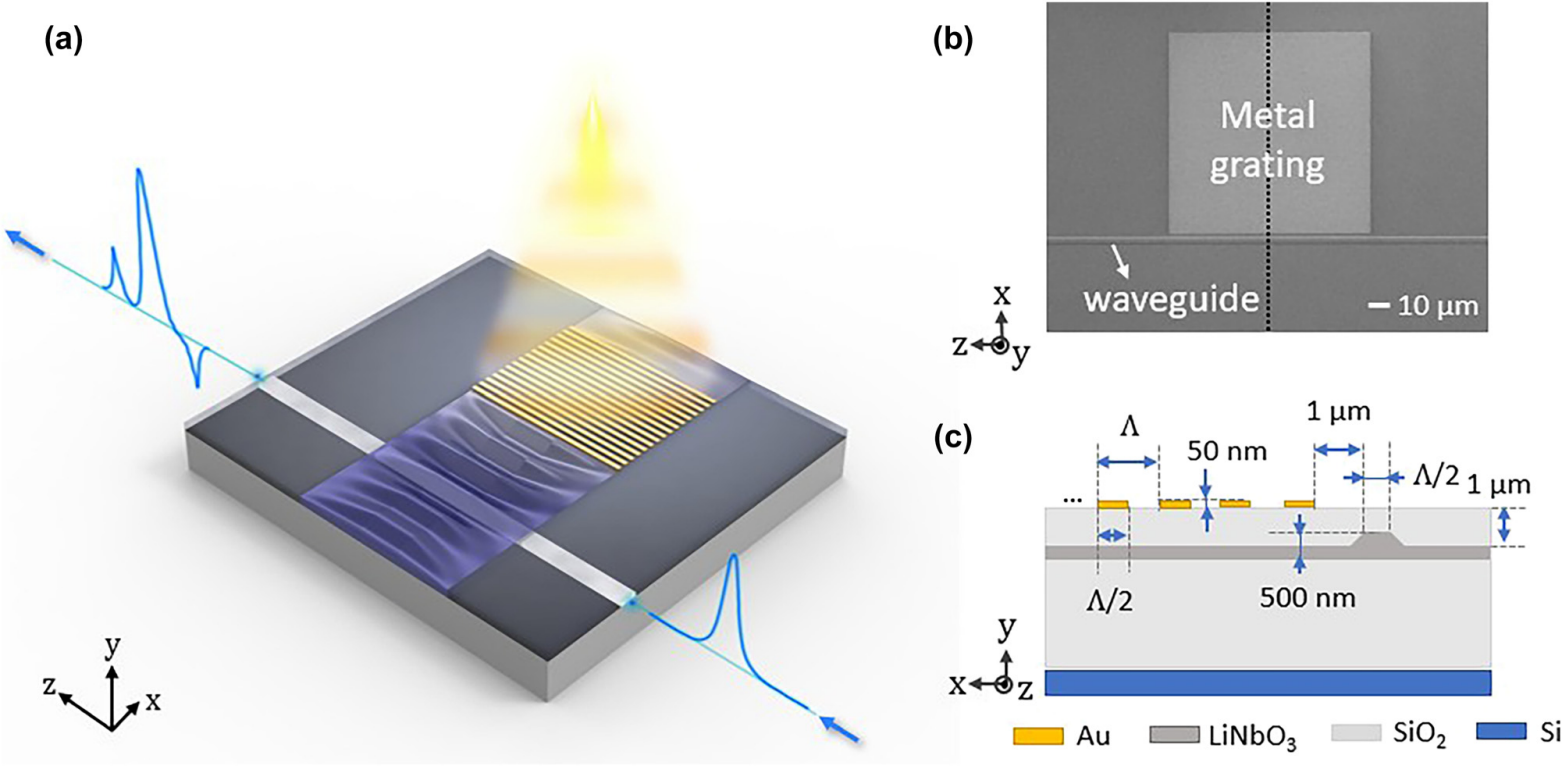
The thermoelastic on-chip acousto-optic modulation device. (a) The conceptual image. Intensity-modulated pump light (orange) illuminates a gold grating, generating a SAW (purple). The SAW induces phase modulation of light (blue) propagating through a nearby optical waveguide. (b) A top-view SEM image of the device. (c) The cross-sectional view of the device along the dotted line in (b). The lithium niobate layer is half-etched to a rib waveguide geometry. The metal grating with a period Λ ranging from 0.4 μm to 3 μm. The dimension is not to scale.

The detailed device configuration is shown in Figure 1. Thermoelastic SAW is generated by a metallic grating illuminated by pump light that is intensity-modulated at a frequency *f*_*m*_ (see Figure 1(a)). The modulated pump light induces a temperature oscillation in the metal grating with an oscillation frequency of *f*_*m*_. The resulting expansion and contraction of the metal induce a periodically varying strain in the silica cladding below the grating. When the product of oscillation frequency *f*_*m*_ and grating period Λ satisfies the SAW velocity, the SAW is built up coherently and propagates along the surface.

Figure 1(b) shows a scanning electron microscope (SEM) image of the chip used in this work, showing the metallic grating (lighter shaded square) and the optical waveguide (the horizontal line). The cross-sectional view of the device along the black dashed line in Figure 1(b) is shown in Figure 1(c). A half-etched TFLN rib waveguide is cladded with a 1-μm thick silica. The metallic grating is deposited on top of the cladding. The thickness of the cladding is chosen to achieve an appreciable spatial overlap between the optical and acoustic waves while maintaining low optical propagation loss. The metallic grating period Λ ranges from 0.4 μm to 3 μm with a duty cycle of 0.5. The waveguide width is half of the grating period to maximize the modulation efficiency [1]. The distance between the metallic grating and waveguide is 1 μm to minimize the attenuation of the acoustic wave when it reaches the optical waveguide, while having a negligible effect on optical propagation loss.

**Figure 2:**
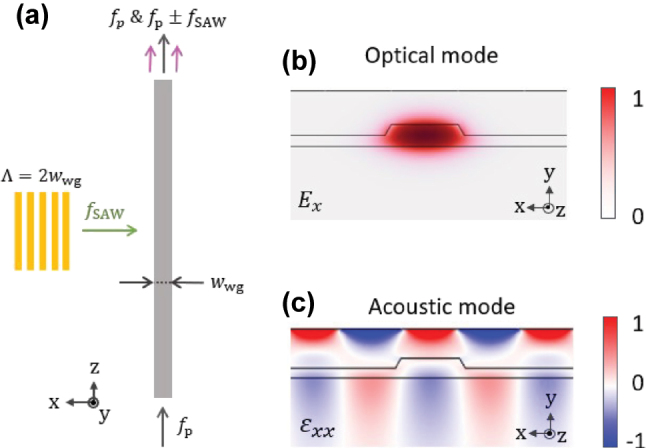
The phase modulation principle. (a) A SAW interacts with the probe light having a frequency of *f*_p_ and generate Stokes and anti-Stokes sidebands at frequencies of *f*_p_ ± *f*_SAW_. Normalized calculated profile of the (b) optical mode and (c) acoustic mode of 1.5 μm width waveguide and 3 μm period metallic grating.

The principle of phase modulation is shown in Figure 2. The optically generated SAW will induce acousto-optic phase modulation of probe light having a frequency *f*_p_ that propagates through a waveguide oriented perpendicularly to the acoustic wave propagation direction (see Figure 2(a)). As a result, a pair of Stokes (*f*_p_ − *f*_SAW_) and anti-Stokes (*f*_p_ + *f*_SAW_) sidebands will be generated, where *f*_SAW_ = *f*_*m*_. The calculated normalized fundamental optical (electric field component *E*_*x*_) and acoustic (strain tensor *ɛ*_*xx*_) mode profiles are shown in Figure 2(b) and (c), respectively. Maximizing the overlap of these modes will enhance the efficiency of the AOM.

The devices are fabricated using a commercially available x-cut lithium niobate on insulator (LNOI) 4-inch wafer (NANOLN), with a 500-nm thick LN thin film. First, SiO_2_ is deposited on LN as an etching hard mask using plasma-enhanced chemical vapor deposition (PECVD). The optical waveguides are then patterned using UV stepper lithography. Next, the exposed resist patterns are first transferred to the SiO_2_ layer using a standard fluorine-based dry etching process, and then to the LN using an optimized Ar^+^-based inductively coupled plasma reactive-ion etching process. The LN is etched with a depth of about 250 nm, leaving a 250-nm thick slab layer. After removal of the residual mask, a 1-μm thick SiO_2_ top cladding is deposited. Then, the gratings pattern is transferred to the photo-resist (PMMA) using electron beam lithography (EBL) and deposited through thermal evaporation with 50 nm gold and following a lift-off process. Gold is chosen because the combination of its heat capacity, thermal conductivity, and thermal expansion induces a near optimal stress in the silica. Finally, the TFLN chips are carefully cleaved with an optical edge coupling loss of approximately 5 dB per facet.

## 3 Experimental setup

**Figure 3:**
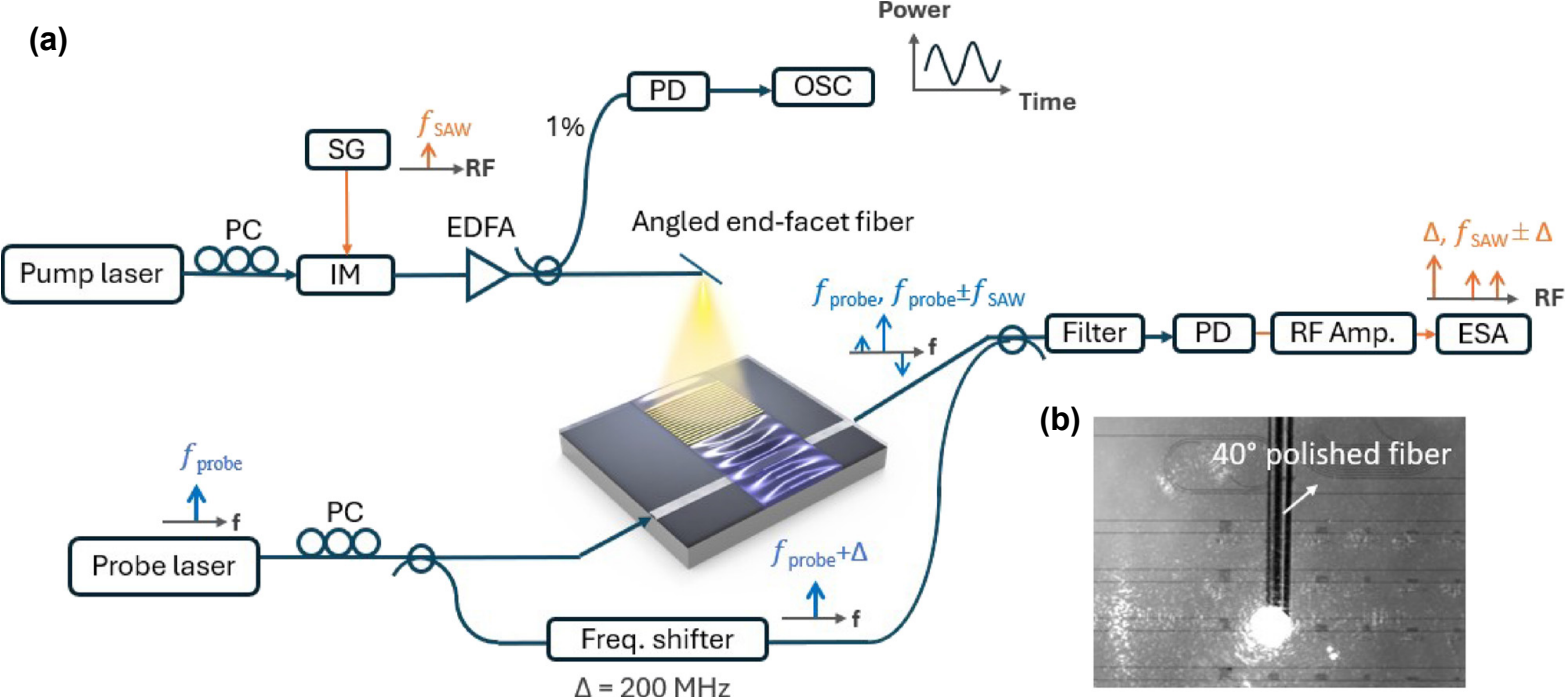
The heterodyne measurement scheme. (a) In the excitation arm, pump light is intensity modulated at a frequency *f*_*m*_ = *f*_SAW_ and is directed toward the metal grating via the end-facet of the fiber, polished at 40°. In the detection arm, probe light is split to the device and the frequency shifter, subsequently combined and filtered to remove any pump light scattered into the path of the probe light. The optical signal is converted to electrical signal via a PD, then amplified by an RF amplifier, and measured by an electrical spectrum analyzer (ESA). (b) The top-view image captured by an infrared camera and showing the metal grating illuminated by the pump light.

The schematic of the measurement setup used to excite and detect the SAW AOM is shown in Figure 3. To excite the SAW, the upper arm contains a pump light (optilab, ULDC-1550-MC, 1,558 nm) modulated by an intensity modulator (IM, Thorlabs, LN81S-FC) and then amplified by an erbium-doped fiber amplifier (EDFA, Amonics, AEDFA-C). The modulation frequency *f*_*m*_ is set by the signal generator (SG, Wiltron, 69147A). A commercially available fiber with the end-facet polished at 40° is utilized to reflect the pump light downward, resulting in the pump light being normally incident on the metallic grating. A top view of this fiber and grating is shown in Figure 3(b), captured by an infrared camera with the pump laser turned on. To monitor the modulation of the pump power after amplification in real time, a power splitter is used to direct 1 % power toward a photo diode (PD, optilab, PD-23-C-DC) connected to an oscilloscope (OSC, Keysight, UXR0134A/B). To detect the phase modulation, we utilize heterodyne measurement to extract the optical power in the generated sidebands. The lower detection arm contains a probe laser (APIC, LN-1550, 1,557 nm) operating at a frequency close to but different from the pump laser, such that scattered pump light into the path of the probe beam can be filtered out. The probe light is partially injected into the optical waveguide to interact with the SAW, and partially sent to a frequency shifter with a upper frequency shift of Δ = 200 MHz. The device and frequency shifter outputs are combined with a 50/50 power combiner. Finally, the combined signal is filtered by a band-pass filter (EXFO, XTM-50) and subsequently detected by a PD (optilab, PD-23-C-DC). The electrical output of the PD is amplified by a radio frequency (RF) amplifier and measured by an electrical spectrum analyzer (ESA, Keysight, N9000B). With this heterodyne detection, the optical sidebands caused by the phase modulation of the probe light are downshifted to the RF frequency domain.

To find the frequencies of interest as measured by the ESA, when a SAW is successfully excited and interacts with the probe optical waves, the light field (*E*_d_) out of the 50/50 power combiner includes following frequency components:

(1) $$\begin{array}{ll} E_{\text{d}} & ∋\sin\left(f_{\text{p}}t\right)\sin\left[\left(f_{\text{p}}+\Delta \right)t\right]-\sin\left[\left(f_{\text{p}}+f_{\text{SAW}}\right)t\right]\\ & \;\times \sin\left[\left(f_{\text{p}}+\Delta \right)t\right]+\sin\left[\left(f_{\text{p}}-f_{\text{SAW}}\right)t\right]\sin\left[\left(f_{\text{p}}+\Delta \right)t\right], \end{array}$$

where, for convenience, the electric field amplitudes are omitted. By using trigonometric angle sum and difference identities, Eq.(1) can be rewritten as:

(2) $$\begin{array}{ll} E_{\text{d}} & ∋\cos\left(\Delta t\right)-\cos\left[\left(2f_{\text{p}}+\Delta \right)t\right]+\cos\left[\left(2f_{\text{p}}+f_{\text{SAW}}+\Delta \right)t\right]\\ & \;-\cos\left[\left(f_{\text{SAW}}-\Delta \right)t\right]+\cos\left[\left(f_{\text{SAW}}+\Delta \right)t\right]\\ & \;-\cos\left[\left(2f_{\text{p}}-f_{\text{SAW}}+\Delta \right)t\right]. \end{array}$$

**Figure 4:**
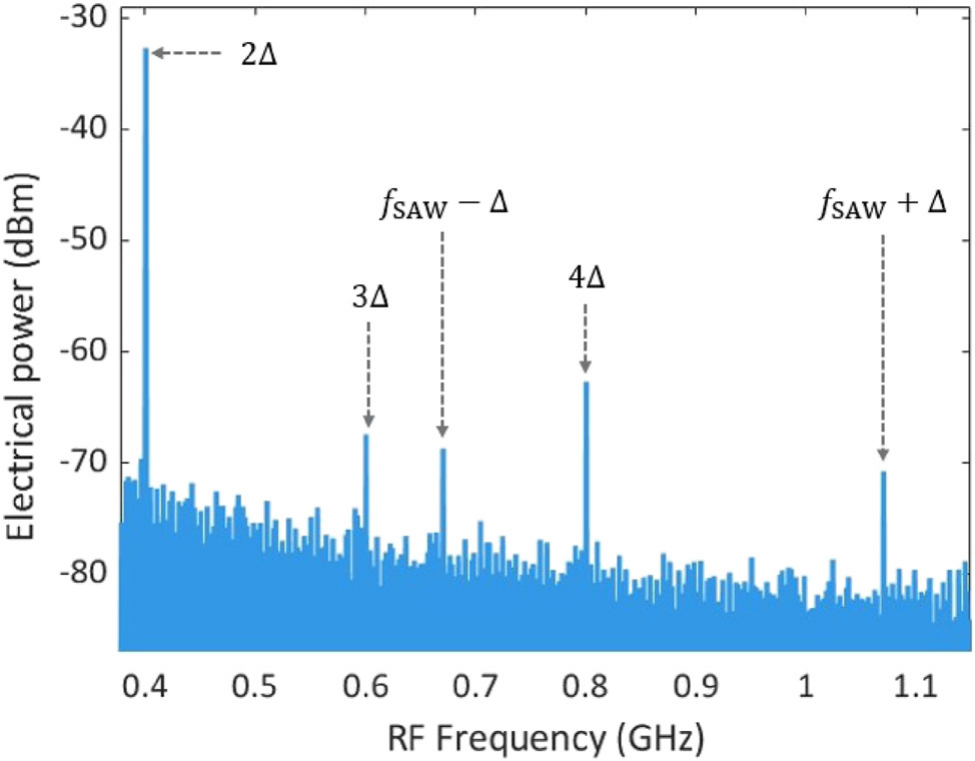
An example the RF spectrum measured by the ESA when a 3-μm period metal grating illuminated by pump light modulated at a frequency of 0.87 GHz. The two lines at *f*_SAW_ − Δ and *f*_SAW_ + Δ are identified with the lower and upper sidebands and prove that a SAW is excited and interacts with the optical probe wave. Other peaks are generated by the frequency shifter. The resolution bandwidth (RBW) is 51 kHz.

Due to the bandwidth of the PD and ESA used, only the frequencies of the frequency shifter Δ, lower sideband *f*_SAW_ − Δ and upper sideband *f*_SAW_ + Δ will be detected. A typical measured RF spectrum is shown in Figure 4 when a 0.87-GHz SAW is excited and clearly shows the two sidebands associated with the SAW. Furthermore, the frequency shifter also generates higher-order signals with the frequency shift of 2Δ, 3Δ, and 4Δ, and these are visible in Figure 4 as well.

## 4 Results

### 4.1 SAW frequencies

**Figure 5:**
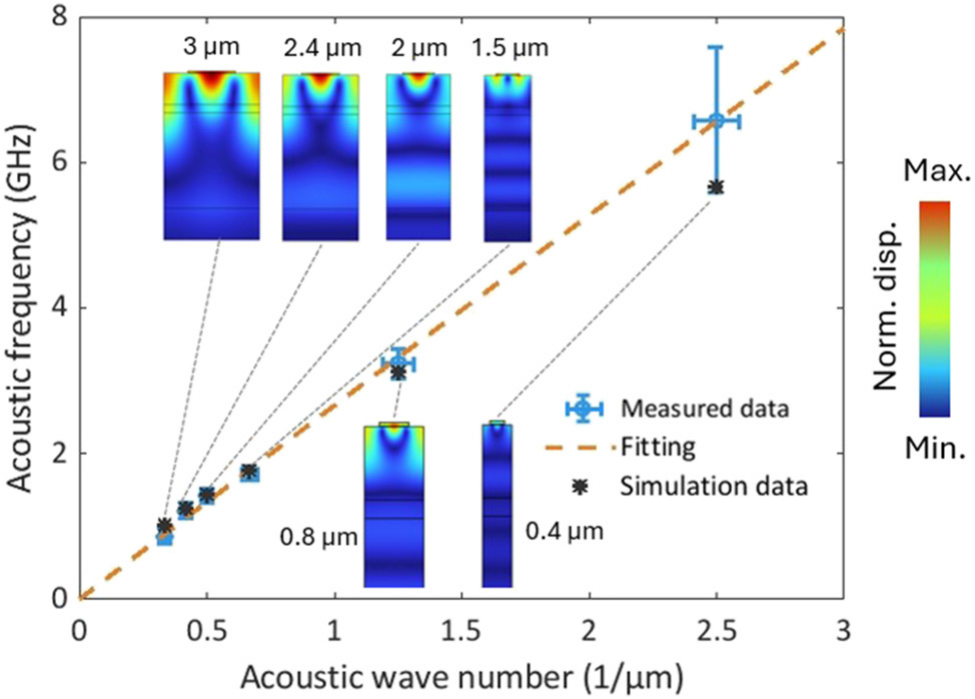
The measured (circle with error bars) and simulated (star without error bars) fundamental SAW frequencies versus the acoustic wavenumber. The error bars indicate the accuracy in the manufactured grating periods and the uncertainty in the measured resonance frequencies. The insets are normalized calculated displacement profiles for different metallic grating periods. The dashed line is a linear fit, $f_{\text{SAW}}=v_{\text{SAW}}\times \frac{1}{\Lambda_{\text{SAW}}}$ , to the data and gives the acoustic velocity equals to (2.8 ± 0.2) × 10^3^ m/s. The acoustic dispersion is neglected here.

The generated SAW frequency in our system is determined by the SAW velocity and the acoustic wavelength. The measured (circle with error bars) and simulated (star without error bars) fundamental SAW frequencies for different metallic grating periods are shown in Figure 5. The insets in Figure 5 show the normalized displacement calculated using the two-dimensional finite element method (FEM). The wave number is inversely related to the grating period, so that the same fabrication error in smaller period leads to a greater error in wave number. We also observed that the uncertainty in the measurement increases with the frequency, because the phase modulation efficiency decreases when frequency increase, causing the signal to blend into the noise level. A higher frequency results in a smaller spatial acousto-optic overlap because the SAW penetration depth is approximately one acoustic wavelength (see insets in Figure 5). Additionally, at higher frequencies, the metallic grating exhibits a reduced temperature oscillation amplitude (see Section 5 below).

The SAW velocity in this 1-μm thick silica top cladding is (2.8 ± 0.2) × 10^3^ m/s, derived from the slope in Figure 5. Compared to bulk acoustic wave (BAW), SAW has a lower acoustic velocity, since the particles are less confined on the free surface compared to bulk acoustic waves. The combination of low acoustic velocity and the fabrication-set smallest grating period limits the frequency of our on-chip AOM to a maximum of several gigahertz, which is also the case for other on-chip AOM [1], [2], [3], [4], [5], [6], [7], [12], [13].

### 4.2 SAW strength

**Figure 6:**
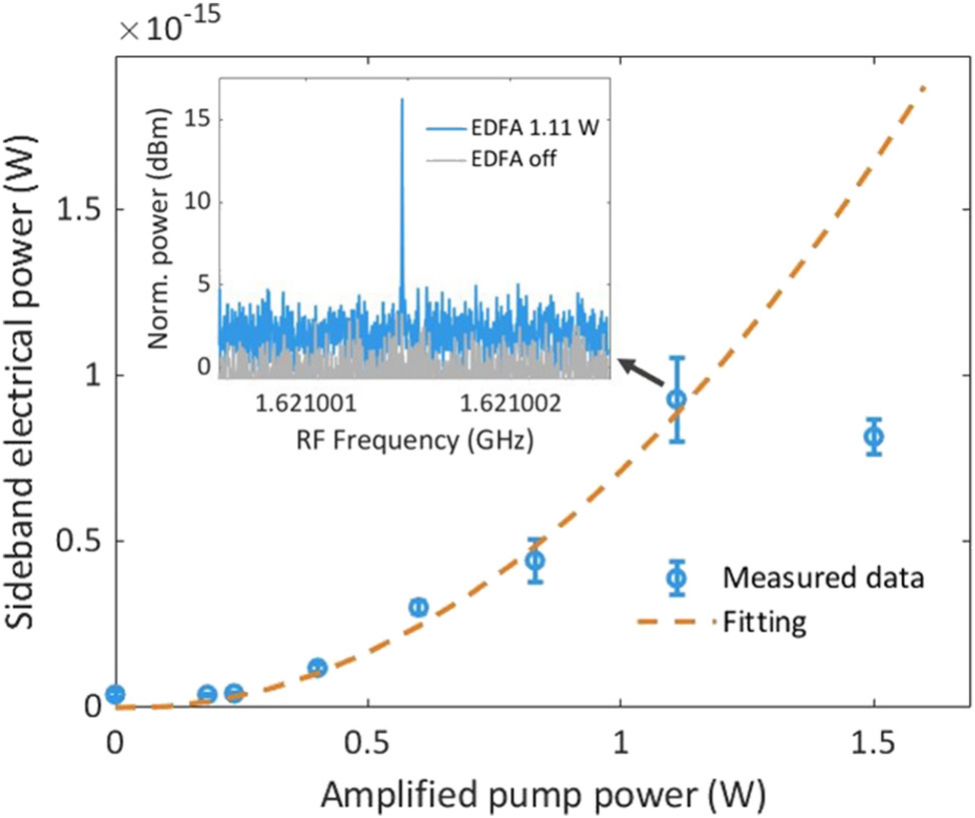
Measured electrical sideband power as a function of EDFA power. A quadratic function is fitted to the data. The inset is an example of an extinction ratio of 15 dB at right sideband, *f*_SAW_ + Δ, between EDFA power at 1.11 W and EDFA turned off. The RBW is 1 Hz. The outlier at 1.5-W EDFA power is due to vibration of the fiber tip caused by the high optical power.

We quantitatively investigate the relationship between the SAW strength and the optical pump power incident on the grating. Figure 6 shows the electrical sideband power, measured using an ESA (see experimental setup on Figure 3), as a function of the amplified pump power. This sideband power is the difference in measured power with the EDFA turned on and off to correct for EMI-induced background at the signal frequency. Since the phase-modulated sideband power *P*_SB_ is proportional to the square of optical pump power, we fit the electrical sideband power to the amplified pump power using a second order polynomial equation, which is shown as the dashed line in Figure 6. The outlier at 1.5 W amplified pump power is not included in the fit, as the high optical power induces a mechanical motion of the fiber tip, which delivers the pump light to the grating.

The on-chip AOM is driven by a thermoelastic SAW, which transfers energy from the pump light to the phase-modulated sidebands. We quantitatively analyze the modulation performance of this thermoelastic acousto-optic modulator. In the experiment, an electrical power *P*_ESA,elec_ at each of the frequencies *f*_SAW_ ±Δ measured by the ESA can be traced back to the on-chip, phase-modulated sideband power as follows. By compensating *P*_ESA,elec_ for the gain of the RF amplifier, *G*_Amp_, we find that the electrical power at the output of the PD, *P*_PD,elec_, at each of the oscillating frequencies is given by

(3) $$P_{\text{PD,elec}}\left(\text{dBm}\right)=P_{\text{ESA,elec}}\left(\text{dBm}\right)-G_{\text{Amp}}\left(\text{dB}\right).$$

Using the responsivity *R*_PD_ of the PD, we can calculate the incident optical power for the beat component between the phase-modulated (anti-)Stokes sidebands and frequency-shifted probe signal as

(4) $$P_{\text{PD,opt}}\left(\text{W}\right)=\left(\sqrt{P_{\text{PD,elec}}\left(\text{W}\right)/R_{\text{L}}}\right)/R_{\text{PD}},$$

where *R*_L_ is the load resistance connected to the PD, which is 50 Ω in the experiment. By correcting for the filter insertion loss, IL_f_ (in dB), we obtain the optical power at the output of the combiner *P*_c,out_ in dBm as

(5) $$P_{\text{c,out}}\left(\text{dBm}\right)=P_{\text{PD,opt}}\left(\text{dBm}\right)+{\text{IL}}_{\text{f}}\left(\text{dB}\right).$$

The optical power at the output of the combiner oscillating at *f*_SAW_ ±Δ (see Eq. (2)) is the result of the beating between the optical sidebands due to phase modulation and the frequency-shifted carrier frequency. Assuming equal optical sideband power, the optical power at each of the frequencies is related to the optical power at the inputs of the combiner as

(6) $$P_{\text{c,out}}\left(\text{dBm}\right)=\frac{1}{2}\left(P_{f_{c}+f_{\text{SAW}}}\left(\text{dBm}\right)+P_{f_{c}+\Delta }\left(\text{dBm}\right)\right),$$

for the beat frequency *f*_SAW_ − Δ, and a similar expression for the other beat frequency. Finally, the modulation depth is defined by the ratio between generated-sideband power and the center frequency power $P_{f_{c}}$ , which, expressed in dB, is given by

(7) $$\eta_{\text{m}}\left(\text{dB}\right)=P_{f_{c}+f_{SAW}}\left(\text{dBm}\right)-P_{f_{c}}\left(\text{dBm}\right).$$

From the experimental data shown in Figure 4, we measure *P*_ESA,elec_ = −70.78 dBm at frequency of *f*_SAW_ + Δ. Using Eq. (3)–(6) and *R*_PD_ = 0.91 A/W, IL_f_ = 4.2 dB and $P_{f_{c}+\Delta }$ = 10.0 dBm, we find that $P_{f_{c}+f_{\text{SAW}}}$ = −84.5 dBm. Using Eq. (7) and $P_{f_{c}}$ = −5.0 dBm, we find that *η*_*m*_ = −79.5 dB. The strength of the refractive index modulation in an on-chip AOM can be normalized to input voltage and device length, which is expressed in terms of *V*_*π*_ *L*_ao_ [1], where *V*_*π*_ is the half-wave voltage and *L*_ao_ is the acousto-optic interaction length. The half-wave voltage can be calculated from modulation depth definition: *m* = *πV*_RF_/*V*_*π*_. In this work, *m* equals to 2.1 × 10^−4^ for an input RF power of 10.5 dBm on an external IM, as shown in Figure 3. As the acousto-optic interaction length *L*_ao_ is 100 μm, we calculate that *V*_*π*_ *L*_ao_ = 2.24 V m (see Supplement for supporting content). Compared to other piezoelectric-based on-chip AOM, this modulation is two orders of magnitude weaker [1], [2], [12]. We also calculate the effective refractive index perturbation Δ*n*_eff_ = 5.2 × 10^−7^ at an average pump power of 1 W. This is only one order of magnitude lower than what has been reported in SOI platform [22], which is reasonable for single-pass AOM in a waveguide compared with multipass in a ring resonator. However, this thermoelastic driven AOM can still be improved significantly (see Section 5 below).

### 4.3 SAW linewidth

**Figure 7:**
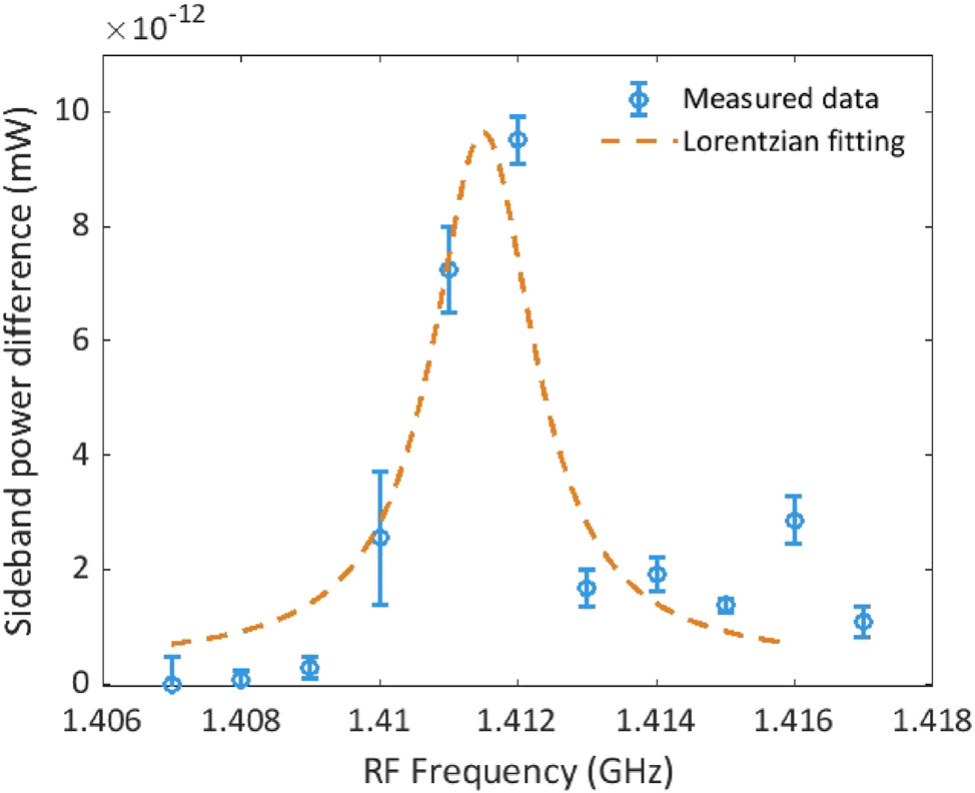
Measured sideband power as a function of frequency for a grating comprising 50 stripes with 2-μm period. The Lorentzian fit provides a FWHM linewidth of 1.8 MHz.

By detuning the signal generator frequency away from the center SAW frequency, the SAW excitation becomes less efficient and the sideband reduces in power. Figure 7 shows the result of such a measurement. By fitting the sideband power with a Lorentzian function, we obtain a full-width at half maximum (FWHM) linewidth of 1.8 MHz when a 50-stripes metal grating with a period of 2 μm at a center frequency of 1.41 GHz. We also measure a linewidth of 2.1 MHz at 0.87 GHz (see Supplement for supporting content). This SAW linewidth is smaller than other values reported for piezo-based AOM [1], [3], [5], [6], which are on the order of tens of MHz. In general, a SAW linewidth is related to several aspects, including scatter losses, uniformity of the grating period, and number of periods. In this work, the uniform gold grating has a small footprint of 100 × 100 μm^2^ on an amorphous silica cladding, which benefits a narrower linewidth compared to other crystalline structures. Additionally, the echoes of acoustic waves reflecting between different interfaces of the layers stack enhance the coupling between SAWs and bulk acoustic waves [25], [26], which might also narrow down the linewidth.

## 5 Discussion

We present the first thermoelastically generated surface acoustic waves in TFLN covered with a thin layer of SiO_2_. The acoustic emitter, which is a metallic grating in this work, transfers an intensity-modulated pump light to a SAW. By exciting a SAW utilizing metallic grating with different periods and varying the pump power, we realize phase modulation at different frequencies and strength, respectively. This acousto-optic modulation technique is compatible with any integrated photonic platform, especially those that are not piezo-active.

The thermoelastic SAW strength is related to several factors, the pump power, the thermal time constant, and the SAW frequency. This thermal dynamics process behaves like a low-pass filter, which means the modulation efficiency will drop for modulation frequency higher than 3-dB bandwidth, which is set by the thermal time constant of the system. The optimization between heat absorption and dissipation decides the temperature oscillation amplitude, Δ*T*. When the metal dissipates heat faster than the period 1/*f*_*m*_ of the modulated pump power, the metal temperature response can fully follow the variations in pump power. However, when metal dissipates heat slower than the modulation period, Δ*T* goes weaker. Therefore, excitation of SAWs and subsequent detection via acousto-optic phase modulation becomes more difficult with increasing SAW frequency (see Supplement for supporting content).

The acousto-optic modulation performance in this device corresponds to a *V*_*π*_ *L*_ao_ of 2.24 V m. Obviously, this modulator’s *V*_*π*_ *L*_ao_ is two orders of magnitude larger than other on-chip AOMs based on the piezoelectric effect [1], [2], [12]. However, there are several ways to improve performance. First, the metal layer thickness could be optimized for maximum absorption, large 3-dB bandwidth, and small mass loading. Given that an optical absorption length, the reciprocal of the absorption coefficient [27], is around 11 nm in gold at 1,550 nm [28], the metal layer thickness can be reduced to 20 nm without affecting the optical absorption and hence the heat penetration. This increases both the amplitude of the temperature oscillation and the low-pass bandwidth of the system. Second, focus the SAW to increase its amplitude by using curved electrodes in the grating [10], or increase the efficiency of heat absorption by replacing the metal electrodes with surface plasmonic resonance nanoparticles [29], [30], [31], which might reduce the acoustic damping as well. Third, place the waveguide inside an acoustic resonator that enhances the amplitude of the acoustic wave, for example, adding a pair of Bragg mirror on both sides. Last but not least, the pump delivery process can be integrated using an additional waveguide layer on the bottom and an optimized grating coupler to direct the pump light toward the metallic grating. A fully integrated three-dimensional photonic–phononic device is expected in the future.

Although this SAW generation is a thermal process, we did not observe thermal crosstalk as a challenge in the experiments. This single-pass phase modulation demonstration should stand for any optical wavelength, including the visible wavelength. However, if we apply this technique in ring resonators or to excite several acoustic emitters at the same time, a space of around 1 mm should be considered in the chip design.

## 6 Conclusion and outlook

In conclusion, we demonstrate for the first time thermoelastic generation of a SAW in silica-cladded TFLN platform without involving the electrical actuation. This technique is also compatible with other low-loss materials without piezoelectricity. Furthermore, thermoelastic AOM is straightforward to implement on both passive and active integrated photonic platform, has promising significant design flexibility and programmable modulation-dependent functionalities that are so-far absent in broader nonpiezoelectric integrated photonic platforms.

## Supplementary Material

Supplementary Material
