# Supplementary material for: Gigahertz thermoelastic acousto-optic modulation in lithium niobate integrated photonic device
Source: Nanophotonics. 2025 Sep 30;14(26):4683–90. doi: 10.1515/nanoph-2025-0252 (PMC12714049; doi:10.1515/nanoph-2025-0252)
Supplement: Supplementary file 1 — Supplementary Material [file j_nanoph-2025-0252_suppl_001.pdf]

1 Supplementary information I: Modulation depth calculation

Here we only consider small signals and only the first order of sideband, the phase-modulated optical spectrum can be written as:

$$E_0 e^{i\omega t + imsin(\Omega t)} \approx E_0 e^{i\omega t}[J_0(m) + J_1(m)e^{ik\Omega t} - J_1(m)e^{-ik\Omega t}], \quad (1)$$

where E_0 is the optical carrier amplitude at the angular frequency of ω, $\Omega = 2\pi f_{\mathrm{SAW}}$ is the angular frequency of acoustic waves, $J_1(m)$ is the first order Bessel function and m is the modulation depth. Figure S1 can be used to convert an experimentally determined ratio $[J_1(m)^2/J_0(m)^2]$ into a modulation depth.

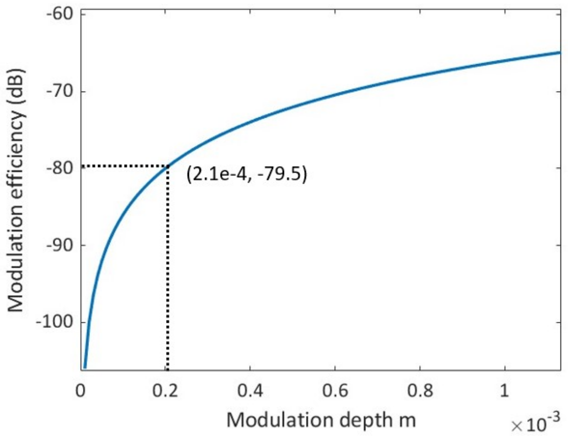

Fig. S1: The relation between modulation efficiency and modulation depth.

2 Supplementary information II: Temperature response

We calculate the temperature responses of a metallic grating with standard discrete calculus method. An example of the calculated temperature response with a pump power oscillating at 0.87 GHz is shown in Figure S2. The rest of setting is listed in Table S1. As we can see, the temperature of the metallic grating will first saturate to an higher temperature compared to surrounding environment and then oscillate around this value. The temperature oscillation amplitude, ΔT, directly contributes to the SAW strength. By increasing the pump power modulation frequency, the temperature oscillation value decreases, as shown in Figure S3, the rest of setting is the same as Table S1. We could conclude that the AOM efficiency decreases at higher SAW frequency. Additionally, in Figure S4, the calculated temperature oscillation amplitude increases when decreasing the metal layer thickness, the rest of setting is the same as Table S1. The metal in this work is designed as a thin layer of 50 nm to enable quick thermal cooling without significantly impacting absorption.

Tab. S1: Example parameters.

Parameters	Number (units)
Metal period	2 μm
Metal width	1 μm
Metal thickness	50 nm
Metal length	100 μm
Metal density (Gold)	19.3e3 kg/m^3
Metal heat capacity	129 $J/(kg * K)$
Number of stripes	50
Thermal conductivity (Gold)	310 $W/(m * K)$
Max/Min pump power	20/1000 mW

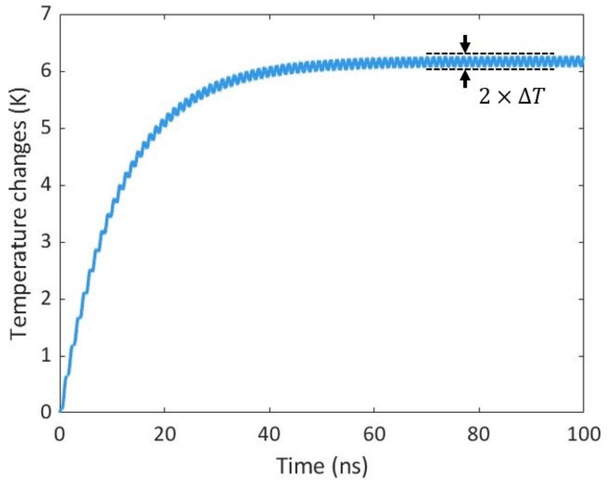

Fig. S2: Calculated temperature responses at 0.87 GHz. The temperature oscillation amplitude ΔT is defined as the oscillation after saturation. The rest of setting is listed in Table S1.

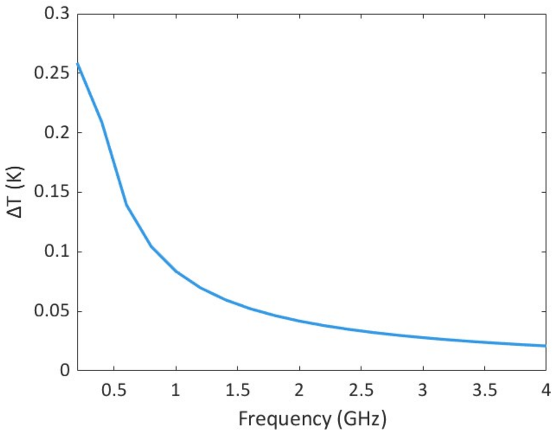

Fig. S3: The relation between calculated temperature oscillation amplitude ΔT and modulation frequency. The rest of setting is listed in Table S1.

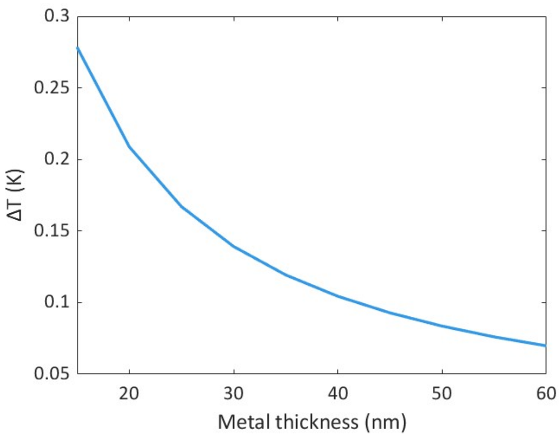

Fig. S4: The relation between calculated temperature oscillation amplitude ΔT and metal layer thickness. The rest of setting is listed in Table S1.

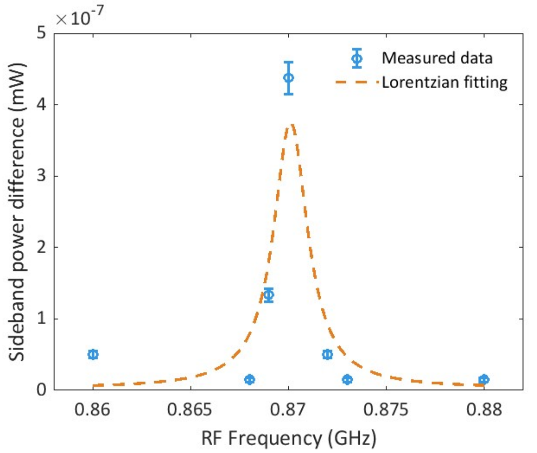

Fig. S5: The relation between calculated temperature oscillation amplitude and metal layer thickness.

3 Supplementary information III: Linewidth at a different frequency

We measure a full-width at half maximum linewidth of 2.1 MHz at a center frequency of 0.87 GHz, as shown in Figure S5. This metallic grating has a period of 3 μm. It has a less number of stripes (33 across 100 μm) compared with 50 stripes of a period of 2 μm in the main text.

We would like to note that this linewidth is narrower than f_{SAW}/N, where N is the number of stripes. We think this narrowing might because that bulk acoustic waves may be generated, after reflection at the substrate, can couple to the SAW [1, 2] and may affect the linewidth.

References

[1] M. Priel, S. K. Bag, M. Slook, et al.,"Thermo-elastic gigahertz-frequency oscillator through surface acoustic wave-silicon photonics", *Opt. Express*, vol.31, no. 1, pp. 684-697, 2022.

[2] L. Dokhanian, S. K. Bag, M. Hen, et al.,"Plasmonic lattice excitation of surface acoustic waves in silicon photonic circuits", *ACS Photonics*, vol.10, no. 6, pp. 066101, 2025.
